# Proangiogenic alginate-g-pyrrole hydrogel with decoupled control of mechanical rigidity and electrically conductivity

**DOI:** 10.1186/s40824-017-0110-x

**Published:** 2017-11-07

**Authors:** Ross J. DeVolder, Yongbeom Seo, Hyunjoon Kong

**Affiliations:** 1 Department of Chemical and Biomolecular Engineering, University of Illinois at Urbana-Champaign, Urbana, IL 61801 USA; 2 Department of Bioengineering, University of Illinois at Urbana-Champaign, Urbana, IL 61801 USA; 3 Department of Pathobiology, University of Illinois at Urbana-Champaign, Urbana, IL 61801 USA

**Keywords:** Alginate hydrogel, Pyrrole, Electrical stimulation, Vascular endothelial growth factor, Elastic modulus

## Abstract

**Background:**

An electrically conductive hydrogel has emerged to regulate cellular secretion activities with electrical stimulation. However, the electrical conductivity of typical hydrogel systems decreases with increasing elastic modulus of the hydrogels because of decreased transport of ions through a polymeric cross-linked mesh.

**Method:**

This study hypothesized that the inverse dependency between electrical conductivity and elastic modulus would be made through the cross-linking of conductive monomer-units conjugated to a hydrophilic polymeric backbone. This hypothesis was examined through the cross-linking of pyrrole groups that were conjugated to an alginate backbone, termed alginate-g-pyrrole.

**Results:**

Hydrogels with increased degrees of pyrrole substitution exhibited a simultaneous increase in the gels mechanical rigidity and electrical conductivity. The resulting hydrogel could control the adhesion and vascular endothelial growth factor secretion of cells via applied electrical stimulation.

**Conclusions:**

This material design principle will be broadly useful to fabricating materials used for various actuation, cell culture, and biomedical applications.

**Electronic supplementary material:**

The online version of this article (10.1186/s40824-017-0110-x) contains supplementary material, which is available to authorized users.

## Background

Over the past several decades, hydrogels have been increasingly used for various biomedical applications including, drug delivery, cell culture, and tissue engineering [1–6]. The successful utilization of hydrogel systems greatly relies on the ability to control their inherent properties, including the mechanical and electrically conductive properties. However, it is still a significant challenge to control these properties [7–9]. For example, the electrical conductivity of typical hydrogel systems is based on the transport of ions through a polymeric cross-linked mesh. Increasing the mechanical rigidity of these system requires increases in cross-linking, which inhibits ion transport, subsequently reducing the electrical conductivity of the system [10–12].

Recently, the incorporation of conductive polymers in hydrogel systems has been used to improve the electrically conductive properties of gels [13–16]. These strategies typically include the diffusion of monomers, such as pyrrole or analine, within a pre-formed hydrogel network, followed by the subsequent oxidative polymerization [17, 18]. This process forms an inter-penetrating (IPN) network consisting of electrically conductive polymers (e.g., polypyrrole or polyanaline) within a polymer cross-linked network. Even though these IPN-structure hydrogels have demonstrated some improved conductive properties, systematical control of both mechanical and electrical properties in a simultaneous manner still remain a challenge. Also, the multi-step processing procedures of these co-networks are inefficient compared to single step hydrogel formation strategies.

Therefore, we hypothesized that an advanced electrically conductive hydrogel system can be formed in a single polymerization step through the cross-linking of conductive monomer-units conjugated to a hydrophilic polymeric backbone. This strategy was examined through the cross-linking of pyrrole groups that were conjugated to an alginate backbone, termed alginate-g-pyrrole. Additionally, we hypothesized that this system could be used to eliminate the inverse dependence between the mechanical and conductive properties of hydrogels. Furthermore, the enhanced conductivity of the gel would stimulate cells adhered to the gel to produce proangiogenic factors more actively, in response to electrical stimuli. This was examined by forming gels using alginate-g-pyrrole with controlled degrees of pyrrole conjugation, which simultaneously controls the cross-linking density and the quantity of conductive polymer within the hydrogel system. Finally, these hydrogels were used in electrical cell stimulation experiments, for controlling various cellular activities.

## Methods

### Materials

Sodium alginate (*M*_w_ ~250,000 Da, FMC Technologies) was provided by FMC Biopolymer. Poly(ethylene glycol) diacrylate (PEGDA, MW 400 g/mol) was purchased from Polysciences. Ammonium persulfate (APS), 2-(N-morpholino)ethane sulfonic acid (MES) hydrate, 1-(2-cyanoethyl)pyrrole (CEP), 2-aminoethyle methacrylate (MA), sodium hydroxide (NaOH), poly(vinyl alcohol), and lithium aluminum hydride (LiAlH_4_) were purchased from Sigma-Aldrich Company (St. Louis, MO). Anhydrous ether was purchased from Mallinckrodt Chemicals. 1-hydroxybenzotriazole (HOBt) was purchased from Fluka (St. Louis, MO). Polydimethylsiloxane (PDMS), curing agents, and silicone glue were purchased from Dow Corning. 3-aminopropyl(diethoxyl)methylsilane and ethylenediamine triacetic acid (EDTA-silane) were purchased from Gelest Industries. Dichloromethane (DCM) and1-ethyl-3-(3-dimethylaminopropyl)carbodiimide (EDC) were purchased from Thermo Scientific. Celite was purchased from Fisher Chemical. Phosphate buffered saline (PBS) was purchased from Cellgro. Vascular endothelial growth factor (VEGF), Mouse Duo VEGF enzyme-linked immunosorbent assay (ELISA), and ELISA reagents were purchased from R&D Systems. Phosphate buffered saline (PBS), Dulbecco’s modification of Eagle’s medium (DMEM), and Penicillin/Streptomycin (P/S, 10,000 U/mL / 10,000 mg/mL) was purchased from Cellgro. Fetal Bovine Serum (FBS) and trypsin-EDTA (0.5%) was purchased from Invitrogen. MTT Cell Proliferation Assay was purchased from ATCC. Type 1 Collagen, Mouse Duo VEGF enzyme-linked immunosorbent assay (ELISA) and ELISA reagents were purchased from R&D Systems.

### Synthesis of N-(3-aminopropyl)pyrrole (APP)

N-(3-amino propyl)pyrrole (APP) was synthesized according to previously reported procedures [19]. Briefly, a solution of 0.2 M 1-(2-cyanoethyl)pyrrole (CEP) dissolved in anhydrous ether (15 mL) was added drop-wise to a suspension of lithium aluminum hydride (LiAlH_4,_ 0.05 mol) in anhydrous ether (150 mL), and the resulting mixture was refluxed for 10 h. After the mixture was cooled, excess LiAlH_4_ was quenched by the addition of water (5.1 mL) and a 15% NaOH solution (1.7 mL). The mixture solution was heated to 40 °C for 2 h and filtered through Celite before evaporating to dryness. Then the water in the mixture was evaporated to collect yellow oil APP and was confirmed through ^1^H NMR (500 MHz, D_2_O).

### Conjugation of pyrrole or methacrylate units to alginate

Sodium alginate was irradiated with γ-rays from a ^60^Co source at a dose of 2 Mrad for 4 h, in order to decrease the M_*w*_ to 100,000 g/mol, as determined through gel permeation chromatography (GPC), as previously reported [20]. Irradiated alginate was dissolved in 0.1 M MES buffer (pH 6.4) at a concentration of 1% (*w*/*v*). Next, APP was added into the alginate solution and stirred at room temperature for 10 min to facilitate a homogeneous dispersion of the pyrrole reagent in the solution. Then 1-ethyl-3-(3-dimethylaminopropyl)carbodiimide (EDC) and 1-hydroxybenzotriazole (HOBt) was dissolved in the reaction solutions and stirred for 18 h. The molar ratio of HOBt, EDC, and APP/AEM was kept constant at 2:2:1. The molar ratio of APP to uronic acids of the alginate was varied from 0 to 0.35. The resulting polymer was then dialyzed with deionized (DI) water for 3 days using a membrane, while replacing it with fresh water every 12 h. The dialyzed polymer solutions were lyophilized and reconstituted to a 7 wt% stock solution with PBS.

The degree of pyrrole substitution to alginate was evaluated by measuring the UV absorbance of the resulting polymers. The alginate polymers with varied substitutions of pyrrole units (alginate-g-pyrrole) were dissolved in DI water at a 0.01% (*w*/*v*) concentration, and the UV absorbance values of the solutions at a wavelength of 210 nm were measured using a CARY 500 Scan UV-Vis NIR Spectrometer. A standard curve, created by measuring the absorbance values of 0.01% (w/v) alginate solutions containing known quantities of APP, was used to determine the degree of pyrrole substitution. Separately, control samples of alginate were conjugated with methacrylic groups following the same procedures as described above and previously [21]. 2-aminoethyl methacrylate hydrochloride (MA) was conjugated to alginate with the molar ratio of MA to uronic acid groups varied from 0.05 to 0.2, and was confirmed using ^1^H NMR (500 MHz, D_2_O).

### Hydrogel preparation

Alginate-g-pyrrole, polyethylene glycol (PEG), and alginate methacrylate hydrogels were prepared through the cross-linking of the pyrrole, acrylate, or methacrylic groups, respectively. Pre-gel polymer solutions were first mixed with a solution of ammonium persulfate (APS) to induce cross-linking. The final polymer concentrations of the alginate-g-pyrrole and alginate methacrylate pre-gel solutions with varied substitutions of pyrrole and methacrylate were 5 wt%, while the concentration of polyethyle glycol diacrylate was varied from 8 to 12 wt%; the final APS concentrations was 0.1 M. Next, in the case of alginate-g-pyrrole and alginate methacrylate, the mixtures were poured between two glass plates separated by 1 mm spacers, and incubated at 70 °C for 1 h. The hydrogels formed between the glass plates were punched into disks with a 0.5 or 1 cm diameter for further characterizations. Additionally, control hydrogels of adipic acid dihydrazide (AAD) cross-linked alginate hydrogels were formed by mixing solutions of AAD with NHS and EDC in a 1:2:2 ratio respectively with the final polymer concentration of 5 wt%. All of the hydrogels were incubated in deionized water (DI), PBS (pH 7.4) or 10× PBS (pH 7.4) at 37 °C for 24 h, exchanging it with fresh media every 4 to 8 h.

### Characterization of hydrogel mechanical properties

The stiffness of the hydrogels was evaluated by measuring a compressive elastic modulus. Following the incubation in PBS for 24 h, gels formed in the shape of disks with a 1 cm diameter and 1 mm thickness were compressed at a rate of 1 mm/min using a mechanical testing system (MTS Insight). The elastic moduli (*E*) of the gels were calculated from the linear slope of the stress (σ) versus strain (є) curve for the first 10% strain. The shear moduli (*G*) were calculated from the linear slope of the stress versus –(*v*-*v* ^−2^) curve, where *v* = 1 - є, for the first 10% strain. In parallel, the degree of swelling (*Q*) of the gels were calculated following:

1 $$ \mathit{\mathsf{Q}}={\mathit{\mathsf{\rho}}}_{\mathit{\mathsf{p}}}\left[\frac{{\mathit{\mathsf{Q}}}_{\mathit{\mathsf{m}}}}{{\mathit{\mathsf{\rho}}}_{\mathit{\mathsf{s}}}}+\frac{\mathsf{1}}{{\mathit{\mathsf{\rho}}}_{\mathit{\mathsf{p}}}}\right] $$

where *ρ*_*p*_ is the polymer density (1.6 g/cm), *ρ*_*s*_is the density of water, and *Q*_*m*_ is the swelling ratio, which is defined as the mass ratio of hydrated gels to dried gels. The cross-linking densities (*N*) were then calculated based on rubber elasticity theory as follows [22]:

2 $$ \mathit{\mathsf{N}}=\frac{\mathit{\mathsf{G}}{\mathit{\mathsf{Q}}}^{\mathsf{1}/\mathsf{3}}}{\mathit{\mathsf{RT}}} $$

where *R* represents the gas constant (8.314 J mol^−1^ K^−1^) and *T* represents the temperature at which the modulus was measured, 25 °C.

### Characterization of hydrogel electrical conductivity

The electrical conductivity of the hydrogels was evaluated by applying a voltage potential and subsequently measuring the current through the gels. Following the incubation in DI water, PBS, or 10× PBS for 24 h, rectangular hydrogels with dimensions of 1.5 cm × 0.5 cm × 1 mm were exposed to a 100 mV alternating potential difference at 1 kHz using an Agilent 33220A Waveform Generator. Simultaneously, the current through the gels was measured using an Agilent 34411A Digital Multimeter. The electrical conductivity (σ) of the hydrogels was determined through the relation:

3 $$ \mathit{\mathsf{\sigma}}=\frac{\mathit{\mathsf{I}}\bullet \mathit{\mathsf{l}}}{\mathit{\mathsf{V}}\bullet \mathit{\mathsf{A}}} $$

where *I* is the measured current, *l* is the length of the hydrogels (1.5 cm), *V* is the exposed potential (100 mV) and *A* is the cross-sectional area of the hydrogels (0.05 cm).

### Preparation of hydrogels for electrical stimulation

Hydrogels were formed within an electrical stimulation platform for future cellular electrical stimulations experiments. The platforms were constructed using PDMS molds adhered to electrically conductive indium tin oxide (ITO) coated glass slides (Sigma Aldrich) (Additional file 1: Figure S1). First, PDMS was poured around 8 mm glass tubes and cured to form a mold with 8 mm wells. Next, the molds were adhered to ITO slides using silicone glue, resulting in a multiple array of wells with electrically conductive ITO well bottoms. A 0.5% solution of either N-(trimethoxysilylpropyl)ethylene diamine triacetic acid or 3-aminopropyl(diethoxyl)methylsilane were added to the wells and incubated for 1 h in order to present reactive carboxylic acid or amine groups on the surfaces of the ITO. The carboxylic acid functionalized surfaces were subsequently reacted with APP in the presence of EDC and HOBt, in excess, resulting in pyrrole presenting surfaces (Additional file 1: Figure S2). Finally, AAD cross-linked alginate and alginate-g-pyrrole hydrogels were formed, as described above, on the amino and pyrrole functionalized surfaces, respectively, forming gels linked to the ITO substrates of the electrically conductive platforms.

### Examination of cellular activities electrically stimulated on alginate-g-pyrrole hydrogels

Fibroblasts (NIH 3 T3) were seeded on hydrogels of alginate-g-pyrrole and AAD cross-linked alginate with similar cross-linking structures in the electrical stimulation platform, exposed to electrical stimulation, and examined for subsequent cellular activities. The hydrogels formed in the stimulation platform were soaked in Type 1 Collage at a concentration of 0.5 mg mL^−1^ for 2 hours before cell seeding. Fibroblasts between passage numbers 10 and 15 were seeded on hydrogels at a density of 1000 or 15,000 cells per well, and were cultured in DMEM supplemented with 10% FBS and 1% P/S at 37 °C. After 24 h, gels with cells seeded at 1000 cells per well were electrically stimulated with a direct potential of 1.0 V for 2 h, by connecting electrodes to the ends of the ITO glass slide of the stimulation platform. Throughout the stimulation period, images were taken at intervals of 1 min and were examined using analytical software (Image J). In parallel, gels with cells at 15,000 cells per well were stimulated with an alternating potential of 1.0 V at a frequency of 10 Hz for 20 min per day for 2 days. Cell media was collected before stimulation (day 0) and after the stimulation period (day 2). The VEGF concentrations in the media were measured using a VEGF ELISA kit, following the manufacturer’s protocol. A calibration curve, prepared by measuring the absorbance values of standards with known concentrations of VEGF, was used to quantify the concentrations of VEGF in the collected cell culture media.

### Statistical analysis

Four to six samples were analyzed per condition unless otherwise specified for all experiments. One-way analysis of variance (ANOVA) was used to determine the statistical significance of data and Scheffe Post Hoc tests were applied to all pair-wise differences between means. Data was considered significant for *p* values <0.05.

## Results

### Alginate-g-pyrrole hydrogels with varied degrees of pyrrole substitution

**Fig. 1:**
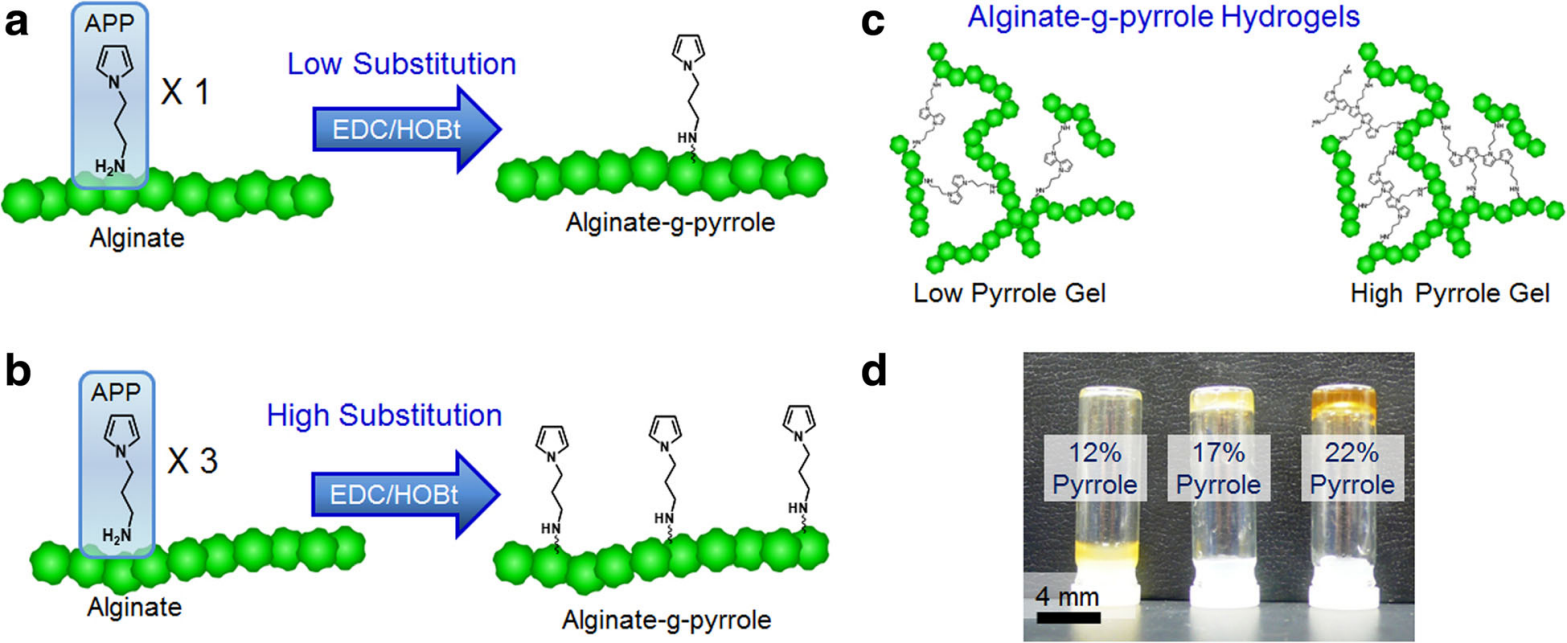
**a, b** A schematic of the pyrrole group conjugation to the carboxylic acid groups of alginate with varied degrees of pyrrole substitution using carbodiimide chemistry. **c** Alginate-g-pyrrole hydrogels formed containing a high and low degree of conjugated pyrrole to alginate. **d** Images of gels formed with varied concentrations of pyrrole substitution to alginate

Pyrrole units were chemically conjugated to alginate using carbodiimide chemistry. First, 1-(2-cyanoethyl)pyrrole was reduced to N-(3-amino propyl) pyrrole (APP) using LiAlH_4_ in ether. Next, varied amounts of APP were attached to alginate by coupling the amine of APP with the carboxylic acid groups of alginate’s uronic acid groups using 1-hydroxybenzotriazole (HOBt) and 1-ethyl-3-(3-dimethylaminopropyl)carbodiimide (EDC) (Fig. 1a & b **)**. By controlling the ratio of APP to uronic acid groups, alginate was conjugated with controlled quantities of pyrrole, and termed alginate-g-pyrrole. The degree of pyrrole substitution to alginate was determined to be 12, 17, 22, 26, and 32%, as evaluated using UV absorbance measurement at 210 nm. In parallel, alginate was chemically linked with varied quantities of 2-aminoethyl methacrylate forming alginate methacrylate with varied degrees of methacrylate substitution: 5, 10, and 15% substitution.

Hydrogels were formed through the cross-linking of pyrrole of alginate-g-pyrrole (Fig. 1c). The addition of ammonium persulfate (APS) to pre-gel polymer solutions of alginate-g-pyrrole with varied degrees of pyrrole substitution, and a subsequent application of heat activated the cross-linking reactions of pyrrole groups, forming gels with controlled pyrrole content. All alginate-g-pyrrole hydrogels were formed with final polymer concentrations of 5 wt%. The activation of alginate-g-pyrrole with a 12% pyrrole substitution did not result in the formation of a rigid hydrogel, because of an insufficient quantity of cross-linked pyrrole necessary to form a gel while alginate-g-pyrrole with higher degrees of substitution formed rigid hydrogel networks (Fig. 1d). Additionally, control hydrogels of polyethylene glycol (PEG), alginate methacrylate, and adipic acid dihydrazide (AAD cross-linked alginate were also prepared. PEG gels were formed by cross-linking varied concentrations of PEG diacrylate (PEGDA) through the addition of APS. Similarly, alginate methacrylate gels with varied substitutions of methacrylate units were formed by cross-linking methacrylate using APS. AAD cross-linked alginate hydrogels were formed through the chemical linking of AAD with the carboxylate groups of alginate through the activated coupling reaction using HOBt and EDC. The final polymer concentration of both alginate methacrylate and AAD cross linked alginate gels was 5 wt%.

### Mechanical and electrical conductivity of hydrogels

**Fig. 2:**
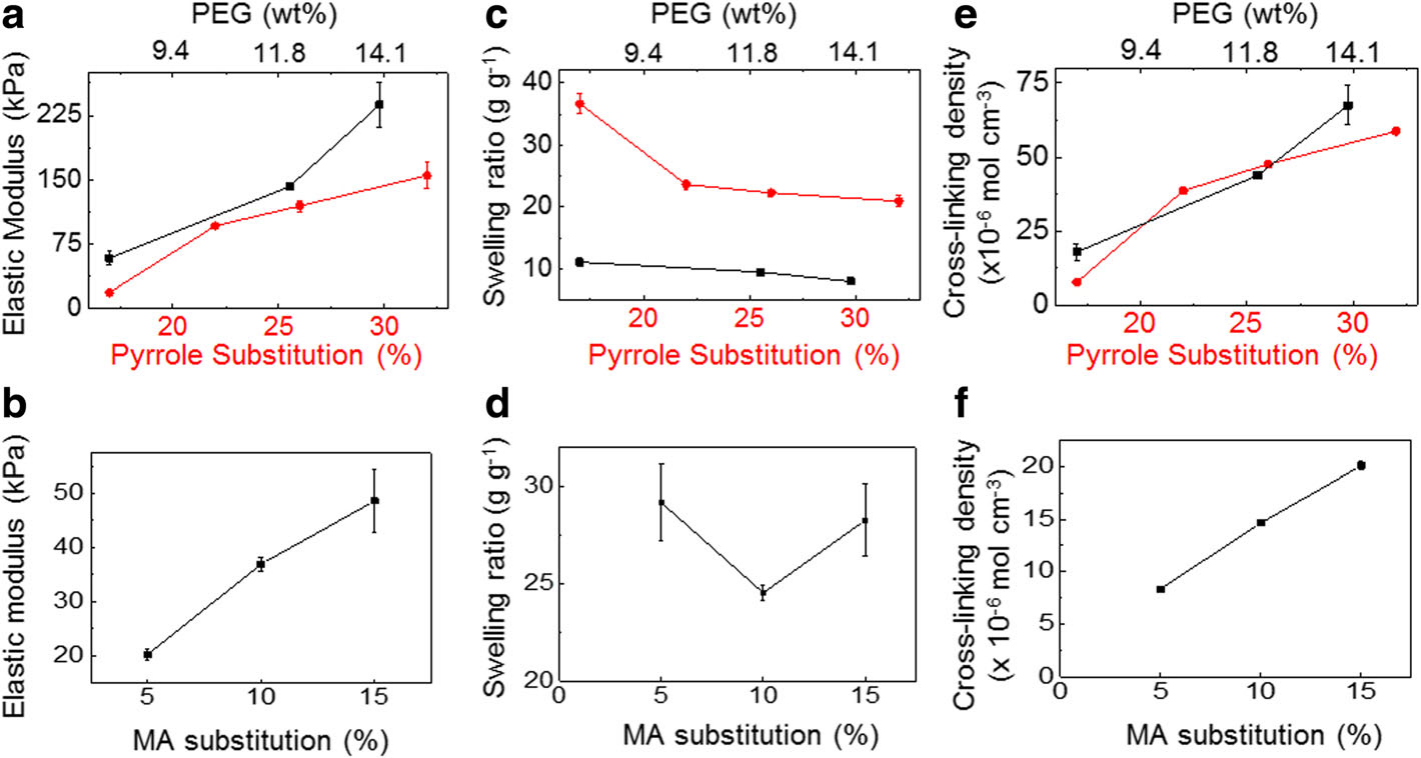
**a, c, e** The compressive elastic modulus (**a**), swelling ratio (**c**), and cross-linking density (e) of alginate-g-pyrrole gels with varied degrees of pyrrole substitution and PEG gels formed with varied concentrations of PEGDA. **b, d, f** The compressive elastic modulus (**b**), swelling ratio (**d**), and cross-linking density (**f**) of alginate methacrylate gels with varied degrees of methacrylate (MA) substitution

The mechanical properties of the hydrogels were characterized through compressive elastic modulus measurements, and the water content of the gels, termed as the swelling ratio, was characterized by measuring the masses of hydrated and lyophilized gels. The elastic moduli of the alginate-g-pyrrole, PEG, and alginate-methacrylate hydrogels were controlled through the degree of pyrrole substitution, the concentration of PEGDA, and the degree of methacrylate substitution, respectively (Fig. 2a and b). The elastic moduli of the alginate-g-pyrrole and PEG gels could be controlled from 0 to 200 kPa. On the other hand, alginate-methacrylate could only be formed with moduli ranging from 0 to 50 kPa, due to the flexible methacrylate linker and solubility limitations of methacrylate substitutions higher than 24%. The swelling ratios of hydrogels decreased with increasing pyrrole substation, PEGDA concentrations, or methacrylic substitutions (Fig. 2c and d). The alginate hydrogels exhibited higher swelling ratios compared to PEG hydrogels due to the charged carboxylate groups of alginate backbone. Finally, the overall number of cross-links for the hydrogel networks, termed as the cross-linking density, was determined using Eq. (2). The cross-linking density for the hydrogels was controlled through varying the degree of pyrrole substitution, the PEGDA concentration, and the degree of methacrylate substitution for the respective hydrogels (Fig. 2e and f).

**Fig. 3:**
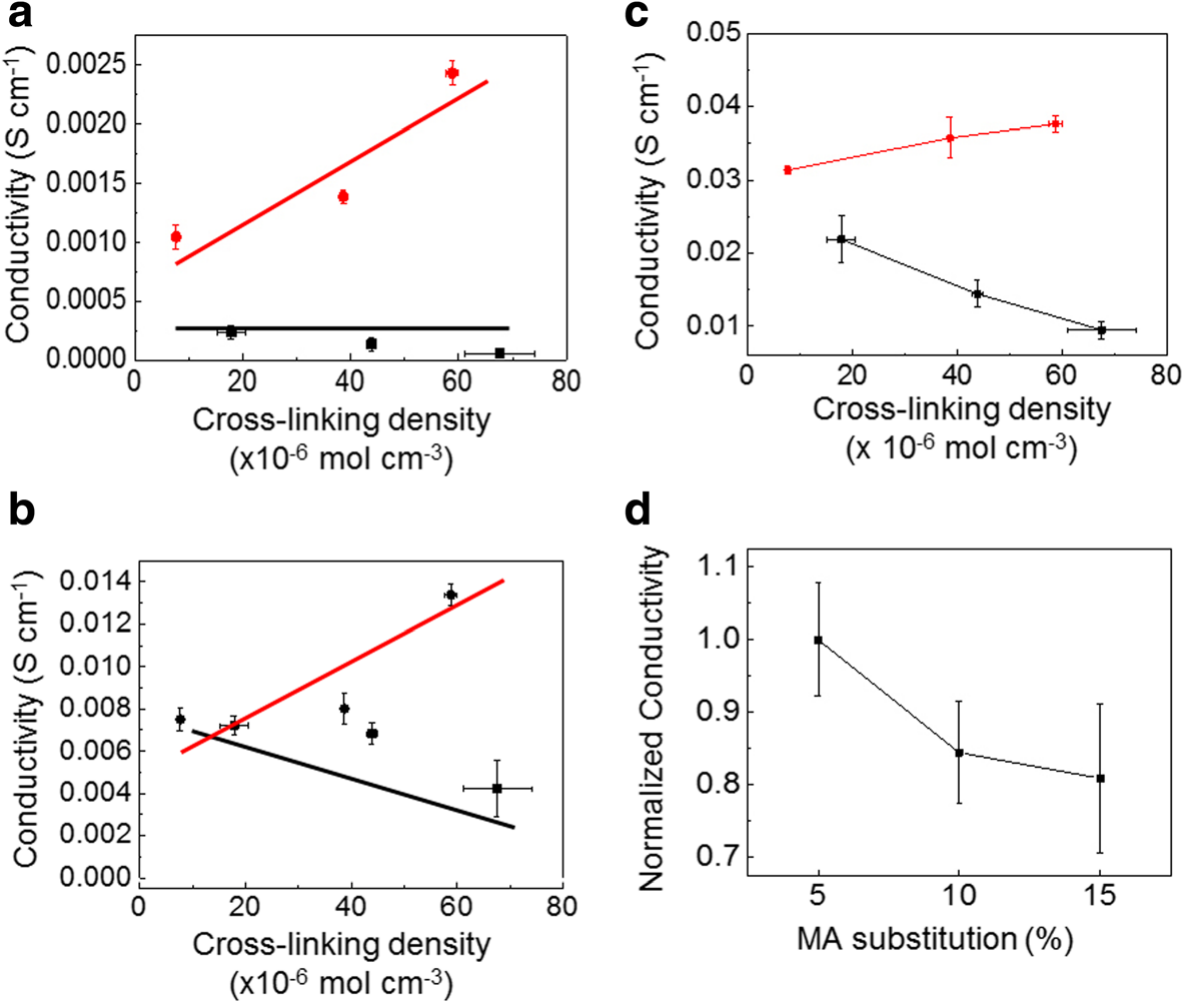
The electrical conductivity of alginate-g-pyrrole (red circle) and PEG (■) gels with varied cross-linking densities in DI water (**a**), PBS (**b**), and 10× PBS (**c**). **d** The normalized electrical conductivity of alginate methacrylate gels with varied cross-linking densities in PBS

On the other hand, the electrical conductivity of the hydrogels was quantified by measuring the current through the hydrogels under a voltage potential. The alginate-g-pyrrole hydrogels exhibited increases in electrical conductivity by increasing the number of cross-links within the hydrogel network in DI water, PBS, and 10× PBS (Fig. 3a and b). On the other hand, PEG and alginate methacrylate gels exhibited decreases in electrical conductivity by increasing the number of cross-links within the hydrogel networks (Fig. 3c and d). Additionally, PEG gels in solutions without any ions exhibited a constant conductivity, independent of the cross-linking density (Fig. 3a).

### Examining the activities of cells adhered to hydrogels under direct potential stimulation

**Fig. 4:**
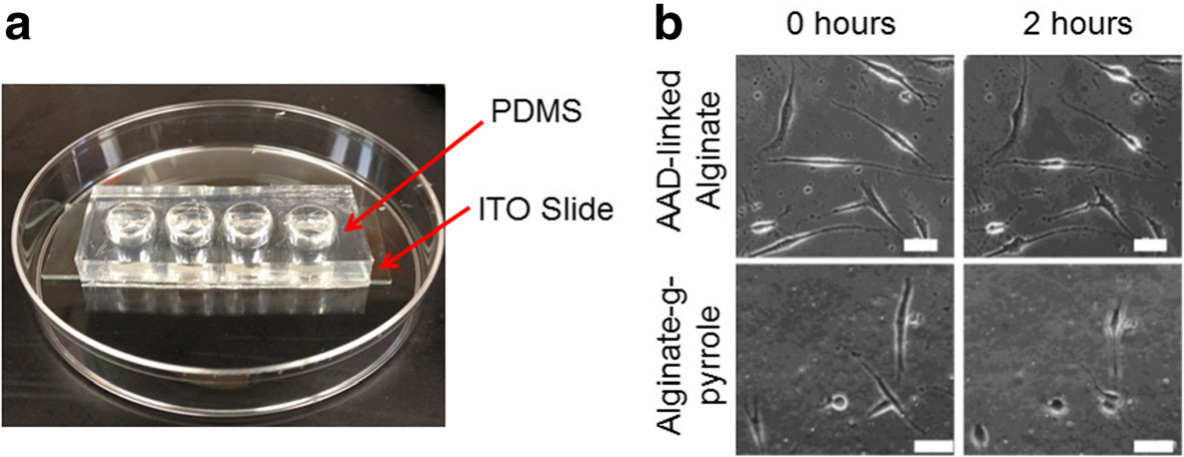
**a** Image of the electrical stimulation platform constructed of PDMS molds bound to ITO slides. **b** Direct potential cell stimulation images of fibroblasts adhered to AAD cross-linked alginate and alginate-g-pyrrole hydrogels after 0 and 2 h of stimulation. The scale bars equal 40 μm

We propose that pyrrole acts as an integrin-binding, synthetic cell adhesion cue as we reported previously [19]. When we tested both alginate and alginate-g-pyrrole hydrogels, we observed that the larger number of cells adhered to the hydrogel of alginate-g-pyrrole than the hydrogel of pyrrole-free alginate methacrylate. The difference was more significant when cells were cultured in media supplemented with low concentration of FBS. The effects of direct potential stimulation on the activities of cells adhered to alginate-g-pyrrole hydrogels were examined using an electrical stimulation platform (Fig. 4a). Alginate-g-pyrrole hydrogels with a 32% degree of pyrrole substitution were prepared in the wells of the electrical stimulation platform. Fibroblast cells were seeded to the gels and then stimulated with potential of 1.0 V over a 2 h period. Additionally, AAD-linked alginate with a similar cross-linking density, but with a 2-fold lower electrical conductivity were used for cellular stimulation experiments (Additional file 1: Figure S3). The cells adhered to the AAD cross-linked alginate gels remained adhered in spread morphology throughout the entire stimulation period, while the cells on the alginate-g-pyrrole retracted their filopodia (Fig. 4b **)**. The stimulated cells on the alginate-g-pyrrole gels remained viable, as demonstrated by a subsequent re-spreading of the cells after the stimulation period.

### Examining the VEGF expression of cells adhered to hydrogels under alternating potential stimulation

**Fig. 5:**
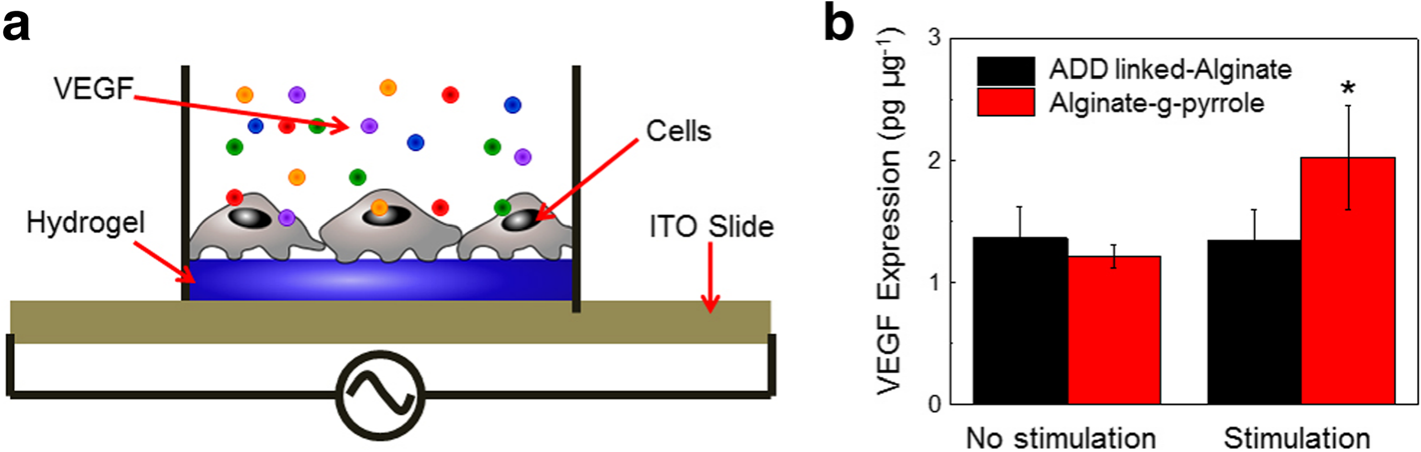
**a** A schematic of cellular endogenous proangiogenic factor expression under alternating current potential stimulation for cells adhered to alginate-g-pyrrole hydrogels in the stimulation platform. **b** The VEGF expression normalized to the overall cellular protein content for fibroblast adhered to AAD cross-linked alginate and alginate-g-pyrrole hydrogels before and after 2 days of alternating potential stimulation

The effects of alternative potential stimulation on the endogenous VEGF expression of cells adhered to alginate-g-pyrrole hydrogels was examined using an electrical stimulation platform (Fig. 5a). Alginate-g-pyrrole hydrogels with a 32% degree of pyrrole substitution were prepared in the wells of the electrical stimulation platform. Fibroblast cells were seeded to the gels and then stimulated with an alternating potential of 100 mV at 10 Hz for 20 min a day for 2 days. Again, AAD-linked alginate with a similar cross-linking density, but with a 2-fold lower electrical conductivity were used (Additional file 1: Figure S3). The stimulated cells on the AAD cross-linked alginate gels exhibited similar VEGF expression levels to that of non-stimulated cells on both AAD cross-linked alginate and alginate-g-pyrrole gels (Fig. 5b). The cell stimulated on the alginate-g-pyrrole gels exhibited a significant increase in the endogenous VEGF expression compared to all other control conditions.

## Discussion

The results of this study demonstrate a strategy to eliminate the inverse dependency between the mechanical rigidity and electrical conductivity of hydrogels by controlling the quantity of electrically conductive cross-linking pyrrole units bound to an alginate polymer, and demonstrate its capacity to manipulate cellular activities including endogenous VEGF expression. The simultaneous increase in the mechanical and electrical properties of the alginate-g-pyrrole hydrogels is controlled through the quantity of pyrrole groups conjugated to alginate, which subsequently determines the quantity of electrically conductive cross-linked pyrrole in the system. Furthermore, the improved electrical properties of the system were demonstrated to have significant impacts on cell adhesion morphology and cellular expression of VEGF for adhered cells, compared to gels with similar mechanical properties, but lower electrical conductivities.

The cross-linking structure of hydrogels significantly impacts their electrical properties by inhibiting ionic transport. Similarly, alginate-g-pyrrole hydrogels formed through the cross-linking of pyrrole groups inhibits ionic transport; however, the cross-linked pyrrole groups provide an additional electron based conductivity, in which electrons transport through the backbone of the polymerized pyrrole. The added electron transport of our system overcomes reductions in conductivity associated with inhibited ion transport, and ultimately increases the conductivity of the system. This is demonstrated by a significant enhancement in electrical conductivity associated with electron transport for the alginate-g-pyrrole gels in DI which contains no ions when compared to PEG gels. Additionally, the similar conductivities of alginate-g-pyrrole and PEG gels with low cross-linking densities in PBS can be attributed to the dominant ionic transport and due to the lack of electron transporting pyrrole groups.

Additionally, the composition of a hydrogel can have a significant impact on the overall conductivity of the system. Hydrogels containing ionic groups have been demonstrated to be more absorbent compared to hydrogels lacking charged groups, which subsequently improves the electrical conductivity of the system via ionic transport. For instance, the alginate-methacrylate and AAD cross-linked alginate that contain negatively charged carboxylate groups have higher conductivities compared to PEG gels without charged groups at similar cross-linking densities. Therefore, increasing the substitution of linkers to alginate to improve the mechanical properties of the system can reduce the overall electrical conductivity by reducing eliminating charged groups. The addition of a conductive linker such as pyrrole can be used to overcome these decreases in electrical conductivity.

The addition of electrically conductive polymers within hydrogel system is a common strategy to improve the electrical properties of hydrogels. These strategies tend to require a two-step polymerization process forming a co-network system. The alginate-g-pyrrole system has the advantage of using an electrically conductive cross-linker to form a gel, which ultimately allows the formation of a single network system, all with a one-step cross-linking process. Additionally, this strategy uniformly distributes the cross-linked pyrrole throughout the gel; whereas other strategies struggle to evenly distribute the electrical conductive polymer throughout the system. The even distribution of pyrrole throughout the alginate-g-pyrrole system, contributes to the overall systematic control of the hydrogel properties.

## Conclusion

This study presents an advanced electrically conductive hydrogel system designed with systematically controllable mechanical and electrical properties. The hydrogels were formed through a single polymerization step by cross-linking conductive pyrrole groups conjugated to alginate with controlled degrees of pyrrole substitution. Hydrogels with increased degrees of pyrrole substitution exhibited a simultaneous increase in the gels mechanical rigidity and electrical conductivity. The alginate-g-pyrrole hydrogels were used to control the adhesion and proangiogenic growth factor secretion of cells adhered to the gels via applied electrical stimulation. We believe that this material design can be extended to a wide array of hydrogel systems, and will be broadly useful for various actuation, cell culture, and biomedical applications.

## Additional file

Additional file 1: Supporting Information. (DOCX 618 kb)

## Abbreviations

AAD: Adipic acid dihydrazide
APP: N-(3-amino propyl) pyrrole
APS: Ammonium persulfate
EDC: 1-ethyl-3-(3-dimethylaminopropyl) carbodiimide
HOBt: 1-hydroxybenzotriazole
ITO: Indium tin oxide
MA: Methacrylate
PDMS: Polydimethylsiloxane
PEG: Polyethylene glycol
PEGDA: PEG diacrylate
VEGF: Vascular endothelial growth factor
