# Supplementary material for: Proangiogenic alginate-g-pyrrole hydrogel with decoupled control of mechanical rigidity and electrically conductivity
Source: Biomater Res. 2017 Nov 7;21:24. doi: 10.1186/s40824-017-0110-x (PMC5678582; doi:10.1186/s40824-017-0110-x)
Supplement: Additional file 1: — Supporting Information. (DOCX 618 kb) [file 40824_2017_110_MOESM1_ESM.docx]

**Supporting information**

**Proangiogenic Alginate-g-Pyrrole Hydrogel with Decoupled Control of Mechanical Rigidity and Electrically Conductivity**

Ross J. DeVolder^1^, Yongbeom Seo^1^, Hyunjoon Kong^1,2,3^*

^1^ Department of Chemical and Biomolecular Engineering, University of Illinois at Urbana-Champaign, Urbana, IL 61801, USA

^2^ Department of Bioengineering, University of Illinois at Urbana-Champaign, Urbana, IL 61801, USA

^3^ Department of Pathobiology, University of Illinois at Urbana-Champaign, Urbana, IL 61801, USA

E-mail: [hjkong06@illinois.edu](mailto:hjkong06@illinois.edu)

**Figures**


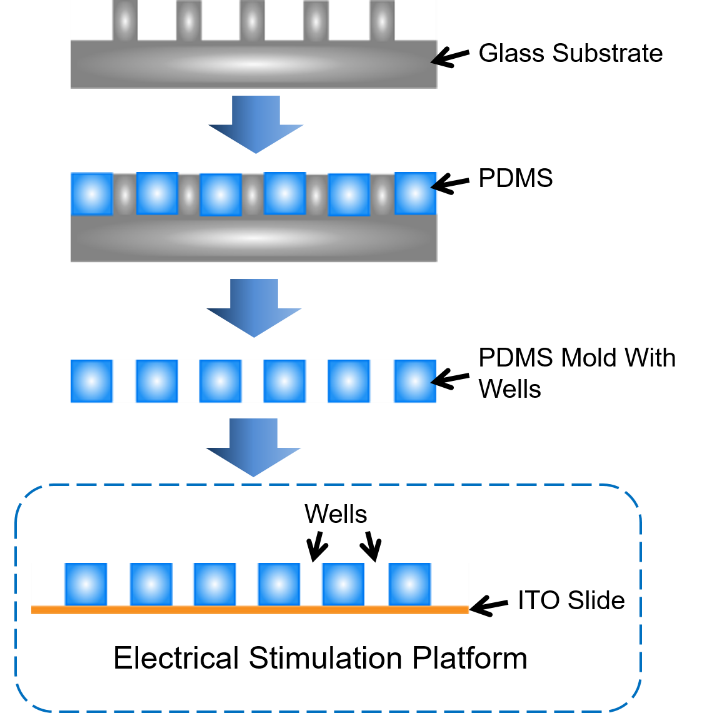


**Fig. S1** Schematic depicting the design of the electrical stimulation platforms. PDMS molds with wells were created and bound to the surface of indium tin oxide slides (ITO), subsequently forming with wells with ITO bottoms.


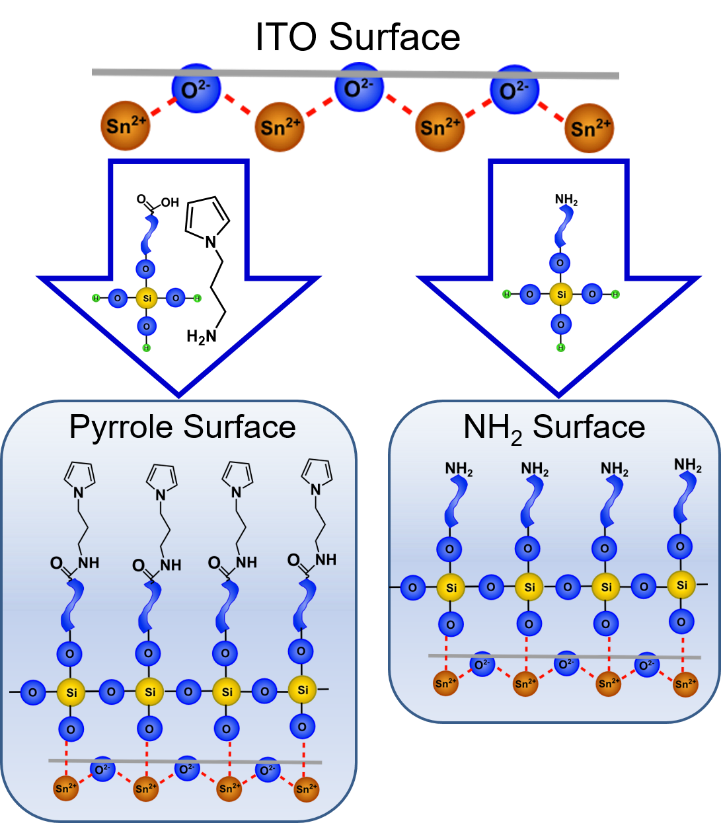


**Fig. S2** A schematic of the surface modification of indium tin oxide (ITO) to present pyrrole (left) and amine groups (right). Pyrrole groups were conjugated to silane coupling agents presenting carboxylic acid groups using carbodiimide chemistry, and silanle coupling agents with amine groups were used to for amine surface modification.


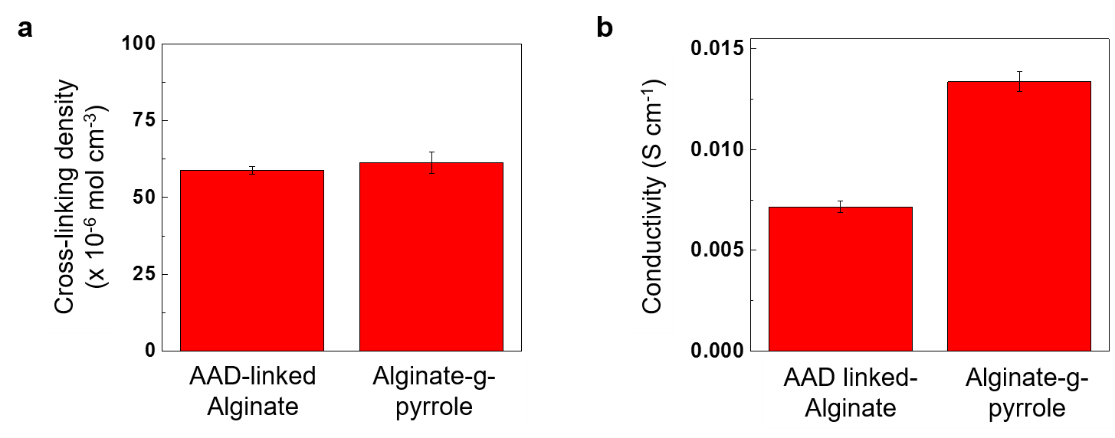


**Fig. S3** The cross-linking density (a) and electrical conductivity (b) of the AAD cross-linked alginate and alginate-g-pyrrole hydrogels used for the cellular electrical stimulation experiments.
